# AI-BLADE toolbox: AI-powered BLADdEr multiparametric MRI analysis for clinical application

**DOI:** 10.1093/bjrai/ubag002

**Published:** 2026-01-22

**Authors:** Muhammad Awais, Ramesh Paudyal, Oguz Akin, Samuel Gold, Stephanie Chahwan, Josip Nincevic, Lisa Ruby, Bernard H Bochner, Jonathan Rosenberg, David Aggen, Hikmat Al-Ahmadie, Alvin C Goh, Lawrence H Schwartz, Amita Shukla-Dave

**Affiliations:** Department of Medical Physics, Memorial Sloan Kettering Cancer Center, New York, NY 10065, United States; Department of Medical Physics, Memorial Sloan Kettering Cancer Center, New York, NY 10065, United States; Department of Radiology, Memorial Sloan Kettering Cancer Center, New York, NY 10065, United States; Department of Surgery, Memorial Sloan Kettering Cancer Center, New York, NY 10065, United States; Department of Radiology, Memorial Sloan Kettering Cancer Center, New York, NY 10065, United States; Department of Radiology, Memorial Sloan Kettering Cancer Center, New York, NY 10065, United States; Department of Radiology, Memorial Sloan Kettering Cancer Center, New York, NY 10065, United States; Department of Surgery, Memorial Sloan Kettering Cancer Center, New York, NY 10065, United States; Department of Medicine, Memorial Sloan Kettering Cancer Center, New York, NY 10065, United States; Department of Medicine, Memorial Sloan Kettering Cancer Center, New York, NY 10065, United States; Department of Pathology, Memorial Sloan Kettering Cancer Center, New York, NY 10065, United States; Department of Surgery, Memorial Sloan Kettering Cancer Center, New York, NY 10065, United States; Department of Radiology, Memorial Sloan Kettering Cancer Center, New York, NY 10065, United States; Department of Medical Physics, Memorial Sloan Kettering Cancer Center, New York, NY 10065, United States; Department of Radiology, Memorial Sloan Kettering Cancer Center, New York, NY 10065, United States

**Keywords:** deep features analysis, model-based analysis, multiparametric MRI, bladder cancer

## Abstract

**Objectives:**

There is a growing need to develop user-friendly, bladder-specific image analysis tools that can produce reliable artificial intelligence (AI)-quantitative imaging biomarkers (QIBs) derived from multiparametric (mp)MRI data for clinical applications. To address it, we developed an AI-powered BLADdEr multiparametric MRI Analysis for Clinical Application (AI-BLADE, current release v1.0) toolbox designed for extracting mpMRI-derived quantitative metrics.

**Methods:**

AI-BLADE is an advanced tool for bladder-specific mpMRI data analysis with 2 core functionalities: (1) Deep Feature Analysis (MRI-DFA toolkit) and (2) Data-Driven Model-Based Analysis (MRI-MBA toolkit). AI-BLADE offers customizable options and serves as a one-stop shop solution for bladder cancer (BCa) clinical applications. The models within DFA and MBA were tested separately on 2 patient cohorts. DFA was used to classify BCa histology subtypes (*n* = 104) with T2-weighted images, while MBA was used to interrogate tumour physiology by deriving mpMRI QIBs, including apparent diffusion coefficient (ADC), and volume transfer constant (K^trans^) obtained from 34 BCa patients.

**Results:**

Out of the 17 AI models tested, the VGG19 model with a decision tree classifier and no feature selection for the fully connected layer 7 achieved the highest area under the curve of the receiver operating characteristic of 0.79 in classifying BCa histology subtypes, demonstrating the strongest performance. The mean ADC and K^trans^ values were 1.22 × 10^−3^ (mm^2^/s) and 0.27 (min^−1^), respectively, reflecting underlying tumour physiology.

**Conclusion:**

The AI-BLADE (v1.0), a flexible and user-friendly software toolbox for analysing mpMRI data, shows strong potential for application in BCa oncology, offering capabilities that can enhance diagnostic accuracy and support improved patient outcomes.

**Advances in knowledge:**

This is the first study to design, develop, and implement a novel bladder-specific AI toolbox for analysing mpMRI data. AI-BLADE enables an advanced image analysis workflow, facilitating AI-QIB-based clinical decision-making for patients with BCa.

## Introduction

The model-based analysis of quantitative multiparametric (mp) magnetic resonance imaging (MRI) data has been accelerated by the availability of software, either by the vendor, in-house developed, or by open-source tools for clinical applications.^1–4^ For example, bladder cancer (BCa) detection and characterization have been improved due to developments in mpMRI, including diffusion-weighted (DW) and dynamic contrast-enhanced (DCE) data acquisition and analysis.^5^ BCa typically originates in the urothelial cells lining the bladder’s interior and is classified as either non-muscle-invasive or muscle-invasive, depending on whether the cancer has invaded the bladder wall.^6^ Accurate assessment of tumour stage, histological subtype, and risk of progression is essential for guiding optimal BCa treatment, including bladder-sparing approaches, neoadjuvant chemotherapy (NAC), or radical cystectomy.^7^ Therefore, a standardized approach for acquisition, interpretation, and reporting, termed VI-RADS (Vesical Imaging-Reporting and Data System), was developed through consensus from existing literature on bladder mpMRI,^8^ including T2w, DW, and DCE images-MRI. However, the VI-RADS scoring system is qualitative; it could be enhanced in the diagnostic accuracy and prognostic value of BCa by incorporating quantitative imaging biomarkers (QIBs) derived from mpMRI data.

Recent advancements in artificial intelligence (AI) for BCa present unparalleled opportunities to improve diagnosis and outcome prediction using the mpMRI data in BCa.^9^^,^^10^ AI architectures, particularly convolutional neural networks (CNNs), have demonstrated remarkable success in enhancing image processing capabilities, with pretrained architectures significantly reducing computation time.^11^ The deep learning models, trained on large datasets, can capture texture and shape details of underlying imaging patterns.^12^ These models have garnered significant attention in medical imaging for their ability to enhance diagnostic accuracy and drive AI-powered advancements in healthcare.^11^ To the best of our knowledge, there is no deep features-based analysis using mpMRI data for BCa to date.

In BCa, model-based mpMRI data analysis is still essential as it helps to derive biomarkers that capture insight into tumour physiology and have shown promise in their clinical application.^5^^,^^7^^,^^13^ In DW-MRI, the image contrast is generated based on the movement of Brownian motion water molecules within tumour tissues.^14^ The apparent diffusion coefficient (ADC) derived from monoexponential modelling of the DW data, assuming a Gaussian nature of water diffusion, reflecting the tissue microstructure and cell membrane integrity, has shown promise in evaluating tumour staging, characteristics, and treatment response in BCa.^15^^,^^16^ The DW data modelling, through an extended non-Gaussian intravoxel incoherent motion model (NG-IVIM), enables the assessment of the deviation of Brownian motion of water molecules from the Gaussian assumption in tumour tissue extracellular space, as well as the diffusion of water molecules within the capillary network.^17^^,^^18^ On the other hand, the time series of contrast agent (CA) kinetic curves obtained from T_1_-weighted DCE-MRI data allows for the evaluation of tumour vascular permeability and microvascular integrity.^19^ The 2 parameters of the Patlak model, a simplified form of the 3-parameter Patlak^20^ or extended Tofts models,^19^ are commonly used for pharmacokinetic analysis of the DCE data that provides estimates of tumour vessel-related parameters. QIBs from these model-based methods are estimated using either linear or nonlinear least squares fitting approaches by minimizing the cost function.^18^^,^^20^^,^^21^ Initial results suggest that fitting DW and DCE data, which were analysed using semi-quantitative or quantitative approaches, hold promise for evaluating bladder tumour staging, aggressiveness, and monitoring the pathological response of NAC or radiotherapy in BCa patients.^7^^,^^17^ For BCa clinical applications, the mpMRI data analyses have been done either using dedicated in-house tools, freely available software, or vendor-provided solutions on standalone advanced workstations.

There is an unmet need for a comprehensive bladder-specific AI toolbox that can process both qualitative and quantitative mpMRI images, derive robust biomarkers for clinical applications, and offer a one-stop-shop solution for BCa clinical applications. This study aims to develop a novel, flexible, vendor-agnostic, and user-friendly AI-powered BLADdEr mpMRI Analysis for Clinical Application (AI-BLADE) toolbox to process bladder mpMRI data, including T2w, DW-, and DCE-MRI, seamlessly for clinical use.

## Materials and methods

### Patient

The institutional review board approved this retrospective MRI study, which is in compliance with the Health Insurance Portability and Accountability Act. Written informed consent was obtained from the patient(s) for publication of this case review, including accompanying images.

A total of 138 patients were enrolled, including 2 cohorts: 104 patients with muscle-invasive bladder cancer (MIBC) and 34 patients with non-muscle-invasive bladder cancer (NMIBC).

### AI-BLADE overall workflow and core functionalities

**Figure 1.**
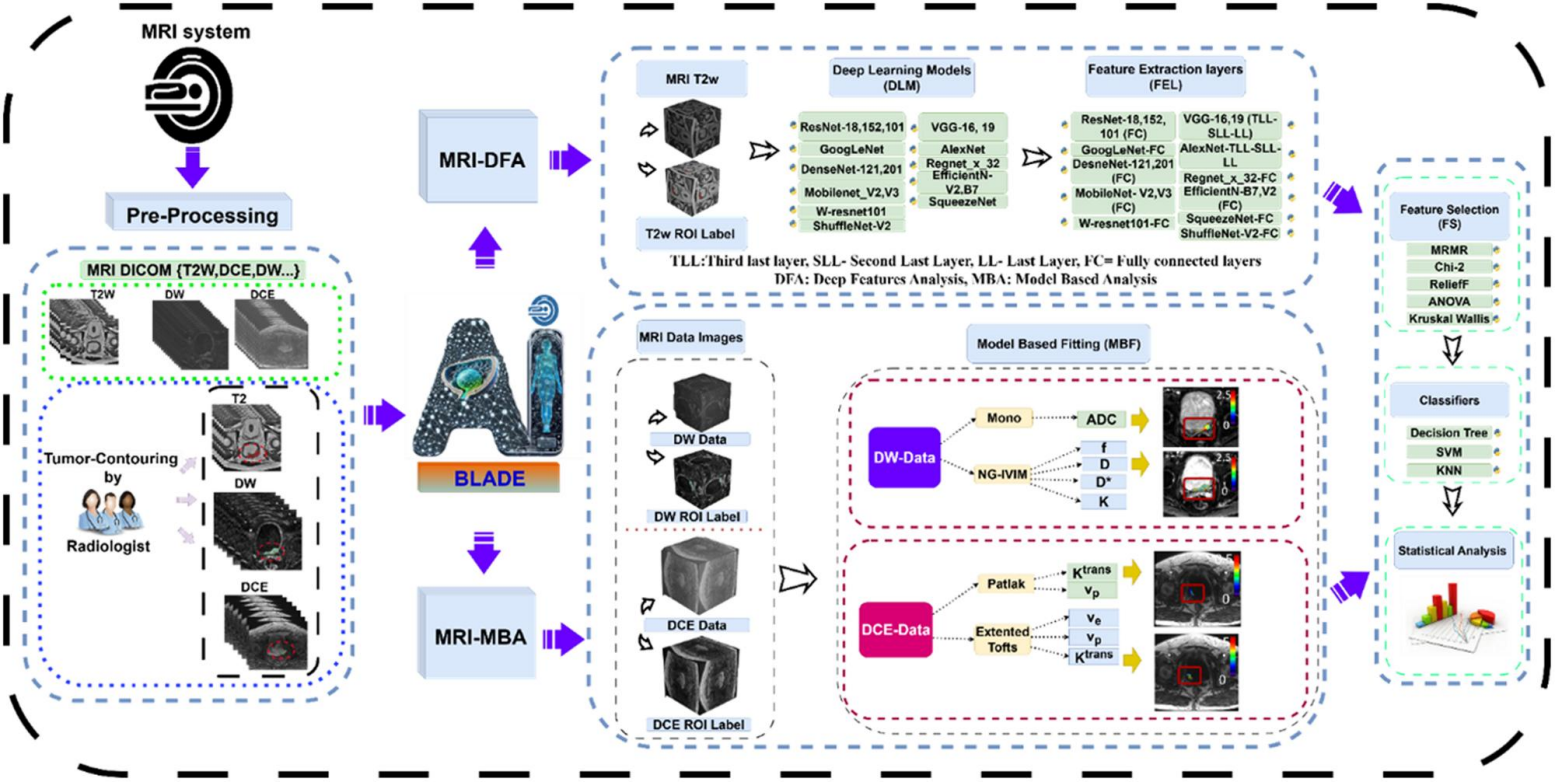
The workflow of the AI-BLADE (AI-powered BLADdEr multiparametric MRI Analysis for Clinical Application) V1.0 toolbox comprises preprocessing steps for multiparametric MRI data. MRI-Deep Feature Analysis (MRI-DFA) consists of a representative T2w image and an annotated region of interest on it, various deep learning models, and their associated feature extraction layers. MRI-MBA (Model-Based Analysis) displays representative diffusion-weighted (DW) and dynamic contrast-enhanced (DCE) images and an annotated region of interest, and parametric maps of apparent diffusion coefficient (ADC × 10^−3^mm^2^/s) and true diffusion coefficient (*D* × 10^−3^mm^2^/s) derived from monoexponential and non-Gaussian intravoxel incoherent motion modelling of DW data, respectively. Volume transfer constant (*K*^trans^ (min^−1^)) maps were generated from Patlak and extended Tofts models from DCE data. Deep features and quantitative imaging biomarkers data are input into the feature selection, classifiers, and statistical analysis, respectively.

**Figure 2.**
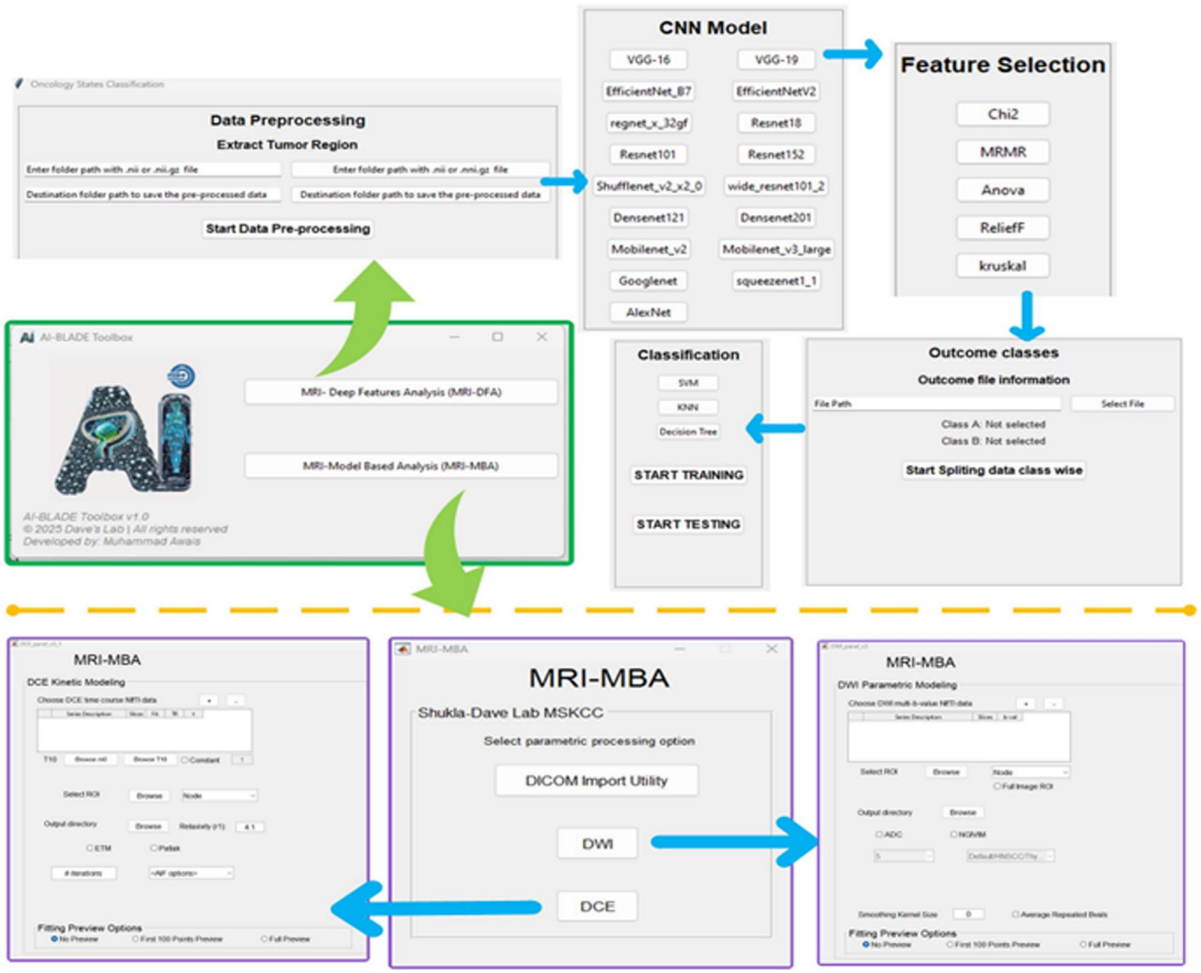
AI-BLADE (AI-powered BLADdEr multiparametric MRI Analysis for Clinical Application) GUIs for the standalone version.

This section describes the implementation of the AI-BLADE toolbox, detailing each step from importing of mpMRI DICOM images to deep feature extraction and computing numerical values from QIB parametric maps, which are obtained through modelling of DW and DCE data. This one-stop-shop solution provided flexibility in testing various configurations, ensuring a thorough assessment of model performance on the given dataset. The AI-BLADE is designed for comprehensive quantitative analysis of mpMRI data. As illustrated in Figure 1, the AI-BLADE workflow provides a streamlined and efficient approach to analysing mpMRI bladder data. Figure 2 shows the graphical user interface, which offers an intuitive, step-by-step process to streamline analysis. This setup is currently being used in an internal active workflow to support ongoing studies. The core functionality of AI-BLADE V1.0 comprises 2 key components: (1) Deep Features Analysis (MRI-DFA), which enables the extraction and analysis of deep features from standard anatomical images, such as T2-weighted(w) MRI images, and (2) Data-driven Model-Based Analysis (MRI-MBA), which allows quantitative analysis of DW and DCEdata. At present, the MRI-DFA is available as a Python-based toolkit, offering flexibility and ease of integration into a variety of imaging data. Meanwhile, the MRI-MBA is a MATLAB-based toolkit (MathWorks, Natick, MA, USA). Both toolkits are designed with advanced, automated tools for accurate, reproducible, and scalable mpMRI data analysis and interpretation.

### Preprocessing steps

AI-BLADE natively supports NIfTI image files and includes an interface for converting DICOM images.^22^ The current toolbox facilitates the integration of mpMRI data, as illustrated in Figure 1 (preprocessing block), including standard T2w, DW-, and DCE-MRI, which are essential for prospective clinical imaging (Appendix Table A1).

Tumour segmentation was performed manually on T2w, DW, and DCE images by an experienced genitourinary radiologist. DW images acquired at *b* = 0 s/mm^2^ and the early-phase image from the DCE series were selected, using T2w images as reference. The segmented regions of interest (ROIs) were stored as labelled masks in NIfTI format, preserving the spatial resolution and alignment with the original images for further processing. To ensure compatibility with the MRI-DFA/MBA toolkits, ITK-SNAP^23^ was used for tumour contouring and saving the NIfTI files. The delineated ROIs from T2w, DW, and DCE images were used as input and integrated into the AI-BLADE platform for subsequent analysis, as described in the sections below.

### MRI-Deep Feature Analysis

The MRI-DFA is a comprehensive, deep learning-based toolkit designed to facilitate the mpMRI data analysis and classification of various clinical endpoints using deep features, such as BCa histology subtypes. This toolkit leverages open-source AI, image informatics libraries, and a statistical toolbox in a modular fashion, providing a streamlined and efficient workflow for medical imaging analysis. There are 3 blocks for MRI-DFA (Figure 1), which include input image, deep learning models, and feature extraction layers, and the rationale behind their design with functionalities is as follows:

#### MRI

It is dedicated to further preprocessing of T2w images, facilitating the conversion of T2w images in NIfTI format into a NumPy format compatible with deep learning models. N4 bias field correction is also performed to mitigate low-frequency intensity non-uniformity on T2w images, as this can distort overall signal intensity.^24^ This block also includes functionality for partitioning data into training and testing sets, ensuring that deep learning models are provided with appropriately formatted and divided datasets. Other key preprocessing steps encompass normalization and resizing of T2w images. Normalization ensures that pixel intensity values are scaled to a consistent range, which is critical for stabilizing the training process of the deep learning models.

#### Deep learning models

This block provides access to a variety of robust deep learning models, each with unique architecture and strengths. The models included in this block are ResNet-18,^25^ AlexNet,^26^ ResNet-152,^25^ VGG-16,19,^27^ ResNet-101,^25^ DenseNet-201,^28^ Swin Transformer,^29^ EfficientNet-B7,^30^^,^^31^ GoogLeNet,^32^ EfficientNet-V2,^31^ EfficientNet-V3,^31^ DenseNet-121,^28^ SqueezeNet,^33^ MobileNet_V2,^34^ MobileNet_V3,^34^ Wide_ResNet101_2,^35^ RegNet_X_32,^36^ and ShuffleNet-V2.^37^ All models are used with pretrained weights, eliminating the need for retraining and allowing for immediate application to new datasets. The inclusion of multiple models provides flexibility, enabling users to select the most appropriate model based on their specific data characteristics and analysis needs. Pretrained models are advantageous because they have been trained on large-scale datasets, learning intricate features and patterns that are transferable to new tasks.^38^

#### Feature extraction layers

This block is responsible for extracting features from the fully connected (FC) layers of the deep learning models. The feature extraction process can be tailored to meet the specific requirements of the user’s dataset, ensuring that the most relevant and discriminative features are captured. The FC layers in deep learning models are particularly valuable because they integrate high-level feature representations learned from earlier convolutional layers. These representations capture complex patterns and dependencies in the data, which are essential for accurate classification or further analysis. Extracting features from the FC layers allows the representations to be used in a compact, fixed-size format that is more suitable for downstream tasks, such as classification or regression. This approach has been widely used in deep learning models to improve the performance of image classification tasks, as the FC layers are designed to map the learned features to decision boundaries.^12^^,^^39^^,^^40^

### MRI-Model-Based Analysis

MRI-MBA is a toolkit designed to perform the quantitative analysis of DW- and DCE-MRI data, enabling the capture of deeper insights into tumour physiology, such as cellularity, tissue microstructure, and blood perfusion and vascular permeability. This toolkit is seamlessly integrated into AI-BLADE to derive QIB metrics from bladder mpMRI data. MRI-MBA consists of 2 blocks (Figure 1), dedicated to further preprocessing of DW- and DCE-MRI data, similar to the MRI-DFA block. MRI-MBA employs data-driven model-fitting approaches to derive QIBs from bladder DW- and DCE-MRI data. The monoexponential and NG-IVIM algorithms were utilized for DW data,^18^ and the Patlak^41^ and the extended Tofts models^19^ for DCE data, respectively.

#### Diffusion-weighted-MRI

DW-MRI generates tissue contrast in images through the diffusion of water molecules.^14^ The degree of DW signal intensity attenuation resulting from diffusion weighting (*b*-value) is modelled using a monoexponential, assuming a Gaussian diffusion, enabling the calculation of ADC values (eqn 1). The NG-IVIM model extends the true diffusion term of the IVIM model by incorporating the diffusion kurtosis coefficient (*K*) (eqn 2), which simultaneously describes water diffusion phenomena in the extracellular space and capillary network.^18^ The NG-IVIM model accounts for deviations of water diffusion from the Gaussian nature.


(1) $$\frac{s_{b}}{s_{o}}=e^{-b\times \mathrm{ADC}}$$

(2) $$\frac{S_{b}}{S_{0}}={fe}^{-b\times D*}+(1-f)e^{-b\times D+\frac{1}{6}K(b\times D{)}^{2}},$$

where *S_b_* and *S*_0_ are the signals with and without diffusion weighting *b* (s/mm^2^) values, ADC (mm^2^/s), *D* (mm^2^/s), and D* (mm^2^/s) are the apparent, true, and pseudo-diffusion coefficients, respectively, and *f* is the perfusion fraction. *K* is the kurtosis coefficient (unitless), a surrogate of the tissue microstructure. These quantitative imaging QIBs are surrogates for tumour cellularity, capillary perfusion, and tissue microstructure.

#### Dynamic contrast-enhanced-MRI

In the DCE-MRI study, T1w dynamic images are acquired before, during, and after CA administration to estimate blood flow and vascular permeability.^19^ Time course of signal intensity data from T1w images is converted to tissue CA concentration (*C_t_*) data using the *T*_10_ and CA longitudinal relaxivity (*r*_1_) value. The tissue longitudinal relaxation rate, *R*_1_ (*R*_1_ = 1/*T*_1_), is expressed as follows under the fast exchange limit approximation^19^:


(3) $$R_{1t}(t)=R_{10}+\;r_{1}\times \;C_{t}(t)\;\to {\;\Delta R}_{1}(t)={\;R}_{1}(t)-R_{10}=r_{1}\;\times C_{t}(t),$$

where *R*_10_ (*R*_10_=1/*T*_10_) is the pre-contrast *R*1 of the tissue. Pre-contrast *T*_1_ value (*T*_10_) values were calculated by fitting the signal intensity data acquired at the 3 flip angles (see Appendix Table A1).

In DCE-MRI, the Patlak and extended Tofts pharmacokinetic models, are frequently employed to analyse DCE data, providing valuable insights into tumour tissue perfusion/permeability and other vascular-related parameters. The Patlak plot, a graphical analysis technique, is based on a unidirectional model for the transfer of CA from the vascular space to the extravascular extracellular space (EES).^42^ This technique uses a linear fit of multiple-time tissue uptake data of the CA to estimate the volume transfer constant (*K*^trans^) and the blood plasma volume fraction (*v_p_*).

The Patlak model is expressed as follows^20^:


(4) $$\frac{C_{t}(t)}{C_{p}(t)}=K^{\mathrm{trans}}\times \frac{\int_{0}^{t}C_{t}(\tau )d\tau \;}{C_{p}(t)}{+v}_{p}$$

The 3-parameter extended Tofts model is given by (eqn 5)^19^


(5) $$C_{t}(t)=K^{\mathrm{trans}}\int_{0}^{t}{e^{-k_{ep}(t-\tau )}\;C}_{p}(\tau )d\tau \;+v_{p}C_{p}(t),$$

where *k_ep_* = *K*^trans^/*v_e_* represents the rate constant of CA transport from the EES to vascular space, *v_e_* and *v_p_* are the volume fraction of the EES and blood plasma space, respectively, and *C_p_*(*t*) is the time course of plasma CA concentration (called arterial input function [AIF]). *K*^trans^, *v_e_*, and *v_p_* are the QIBs that reflect tumour perfusion and permeability, leakage space of CA, and vascular integrity.

For this study, as a clinical test case, we used AI-BLADE’s MRI-MBA to generate parametric maps and extract numerical values from DW, and DCEMRI-derived QIBs described in the above model equations, which are the surrogates of tumour cellularity, tissue microstructure, and vascular perfusion/permeability. MRI data were acquired using standard methods,^43^ including DW and DCE imaging protocol, from patients with 34 NMIBC.

The final analysis for clinical endpoints is achieved by using 3 blocks, which are briefly detailed here.

### Feature selection

Once deep features from MRI-DFA and/or numerical values from the QIBs parametric maps generated using the MRI-MBA toolkits are extracted from the respective models, they will be used for feature selection. The feature selection block includes several algorithms for selecting the most relevant features, including Minimum Redundancy Maximum Relevance (MRMR) and ReliefF,^44^ chi-squared (Chi-2),^45^ ANOVA (analysis of variance),^46^ and Kruskal-Wallis^47^ algorithms. Feature selection helps reduce the dimensionality of the data and improves the efficiency and performance of the classification models by focusing on the most informative features.

### Classification

Classification has multiple classifier options to perform the final classification, from bladder mpMRI data that includes tumour detection, identifying histology subtypes, and risk stratification for the prediction of treatment response and outcome. By offering various classifiers, the toolbox allows users to choose the best-suited method for their specific analysis, enhancing the robustness and accuracy of the classification results. The classifiers include traditional machine learning algorithms such as Support Vector Machines (SVM),^48^ Decision Tree,^49^ and k-Nearest Neighbors (k-NN).^50^

### Statistical analysis

A final component of the workflow plays a pivotal role in providing a statistical evaluation of the classification model’s performance. It calculates key metrics, including the receiver operating characteristic (ROC) area under the curve (AUC), accuracy, sensitivity, specificity, precision, and the F1 score. These metrics collectively offer a thorough and nuanced assessment of the model, addressing its ability to differentiate between classes, handle imbalances, and maintain reliability in predictions. The inclusion of the ROC curve, which plots the true positive rate against the false positive rate, provides a visual and quantitative measure of the model’s discriminatory power across various thresholds. By reporting these statistical parameters, it ensures that the classification results are reliable and interpretable, aiding researchers and practitioners in making informed decisions based on the model’s output. Here, we are presenting the classification results for BCa histology subtypes using the ROC curve to showcase the model’s robustness and overall effectiveness.

## Results

AI-BLADE is currently being used to analyse mpMRI data for the BCa.

### MRI-Deep Feature Analysis

For this study, as a clinical test case, the AI toolbox, AI-BLADE, was used to extract deep features from MRI-DFA for classifying BCa histology subtypes. MRIs were acquired using standard imaging protocol,^43^ and data from 104 MIBC patients (median age: 66 years; 66 M, 38 F) were included. The pathology evaluation classified 65 patients as pure urothelial carcinoma (UC) and 39 patients as non-predominant variant histology (npVH). Deep features were extracted from 4 AI models for this analysis.

### Final analysis

The performance of these models was evaluated using the AUC metric. Higher AUC values, closer to 1, indicate stronger discriminatory power, meaning the model is more effective at differentiating between PureUC and npVH. The final results for the 4 AI models are as follows:

#### ResNet152

**Figure 3.**
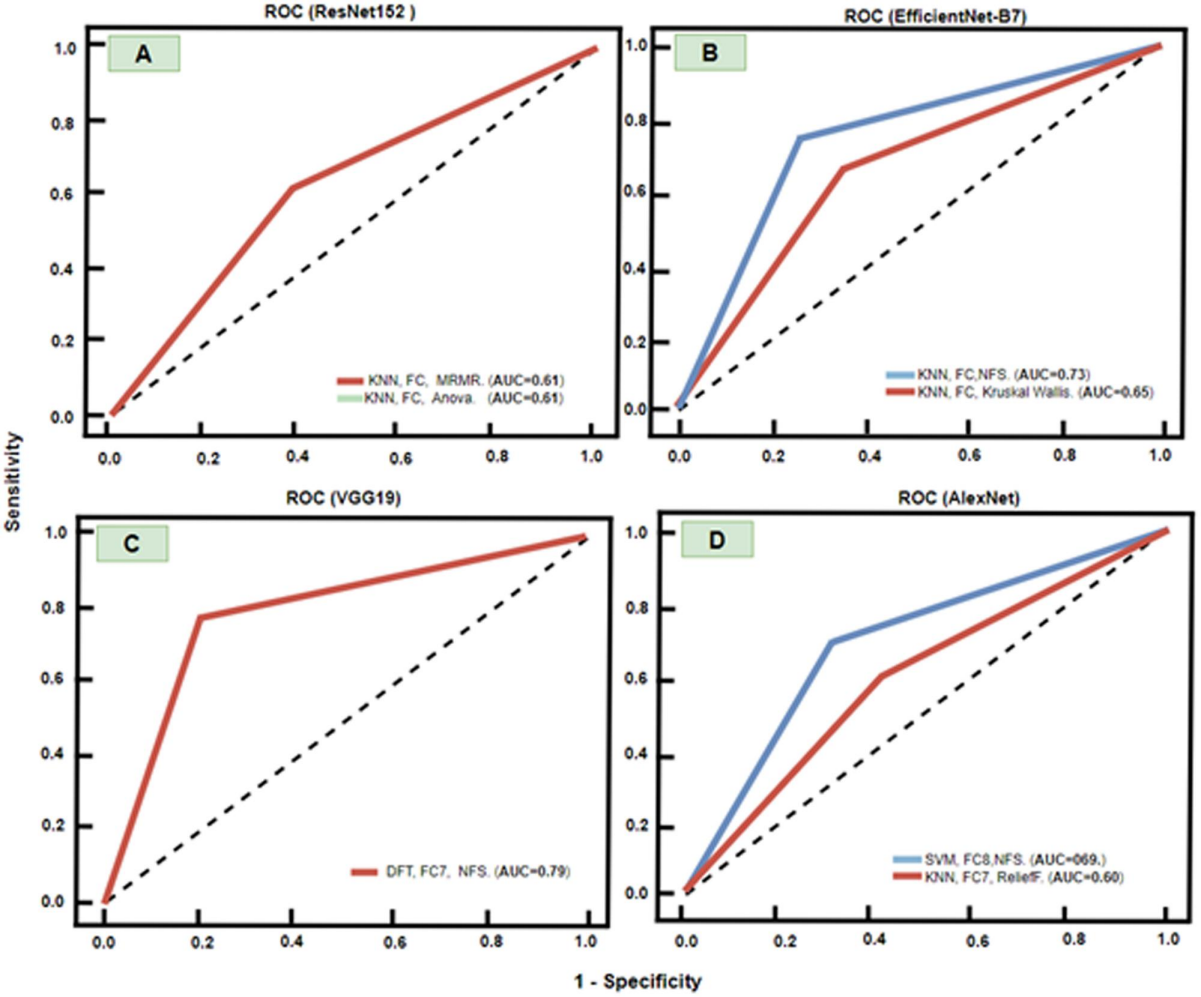
ROC curve for AI models with different feature selection algorithms and classifiers. (A) ROC curves for ResNet152, (B) ROC curves for EfficientNet-B7, (C) ROC curves for VGG19, (D) ROC curves for AlexNet. Abbreviations: DFT = Decision Fine Tree; FC = fully connected layer; NFS = no feature selection; ROC = receiver operating characteristic.

The ResNet152 model in the MRI-DFA toolkit with the KNN classification algorithm was evaluated using 2 feature selection techniques: ANOVA and MRMR. The model utilized an FC layer for classification. Regardless of whether ANOVA or MRMR feature selection was applied, the ResNet152 model consistently yielded an AUC value of 0.61, as shown in Figure 3A. This result suggests that the choice of feature selection method did not significantly affect the model’s performance.

#### EfficientNet-B7

Using multiple settings from our MRI-DFA toolkit, deep features were extracted from the EfficientNet-B7 model, and the KNN classifier was employed with Kruskal-Wallis feature selection. This configuration resulted in an AUC of 0.65, which is lower compared to the AUC of 0.73 achieved with the Decision Tree classifier and no feature selection, as shown in Figure 3B.

#### VGG19

Using the MRI-DFA toolkit settings with the VGG19 model, the Decision Tree classifier, and no feature selection for FC7, an AUC of 0.79 was achieved, as shown in Figure 3C. This was the highest AUC among all models tested, indicating strong performance in distinguishing between the npVH and Pure UC classes in the dataset.

AlexNet from the MRI-DFA toolkit settings with the AlexNet model, the SVM classifier, and an FC (FC7) layer without feature selection achieved an AUC of 0.69. When the KNN classifier was used with the FC7 layer and ReliefF feature selection, the AUC decreased to 0.60, as shown in Figure 3D. This suggests that while the feature selection method was applied, it did not enhance the model’s performance in this case and, in fact, resulted in a lower AUC. The MRI-DFA toolkit allowed comprehensive evaluation of these models across multiple settings and algorithms, including SVM, KNN, and decision tree classifiers.

A detailed comparative analysis of all configurations using the additional pretrained models integrated within the MRI-DFA framework is provided in Appendix Figure A1. This table serves as a reference for model benchmarking and highlights the relative contribution of each network to the MRI-DFA toolkit’s classification capability.

### MRI-MBA-derived QIBs

MRI-MBA toolkit is seamlessly integrated into AI-BLADE to derive QIBs from NMIBC patients’ mpMRI data, which facilitates the quantitative analysis of DWand DCEdata, enabling deeper insights into tumour physiology. DCE data were acquired with a temporal resolution of 5 s/phase, and a total of 35 phases, and AIF was extracted as detailed elsewhere.^51^ The mean *T*_10_ and *r*_1_ values of 1.16 (s) and 3.9 mM^−1^s^−1^ were used for the analysis. The extracted QIBs ADC, *K*, and *K*^trans^ are the surrogates of the tumour cellularity, tissue microstructure, and vascular permeability.

**Figure 4.**
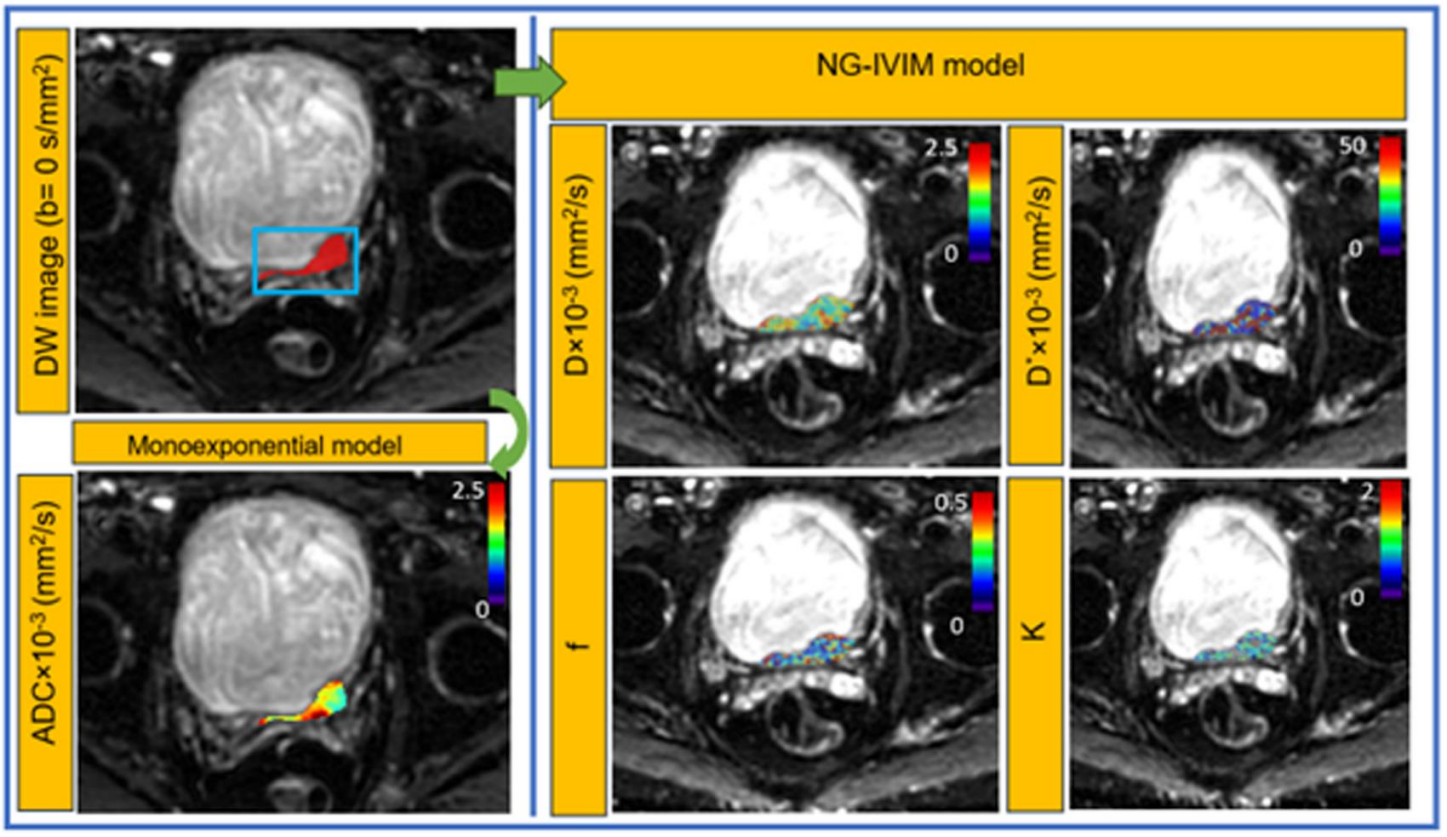
MRI-MBA (Model-Based Analysis) provides fitting algorithms to analyse diffusion-weighted (DW) data using monoexponential and non-Gaussian intravoxel incoherent motion (NG-IVIM) models. The models derived representative parametric maps are overlaid on the diffusion-weighted images (*b* = 0 s/mm^2^).

Figure 4 shows the representative parametric maps generated from the MRI-MBA toolkit for DW data, which utilized both the monoexponential and NG-IVIM models. The mean (±SD) values of ADC, *D*, D*, *f*, and *K* values were 1.22 ± 0.32 × 10^−3^ (mm^2^/s), 1.21 ± 0.37 × 10^−3^ (mm^2^/s), 25.57 ± 8.57 × 10^−3^ (mm^2^/s), 0.24 ± 0.06, and *K* = 0.41 ± 0.16, respectively, were obtained from patients with pure UC of NMIBC (*n* = 22) prior to treatment.

**Figure 5.**
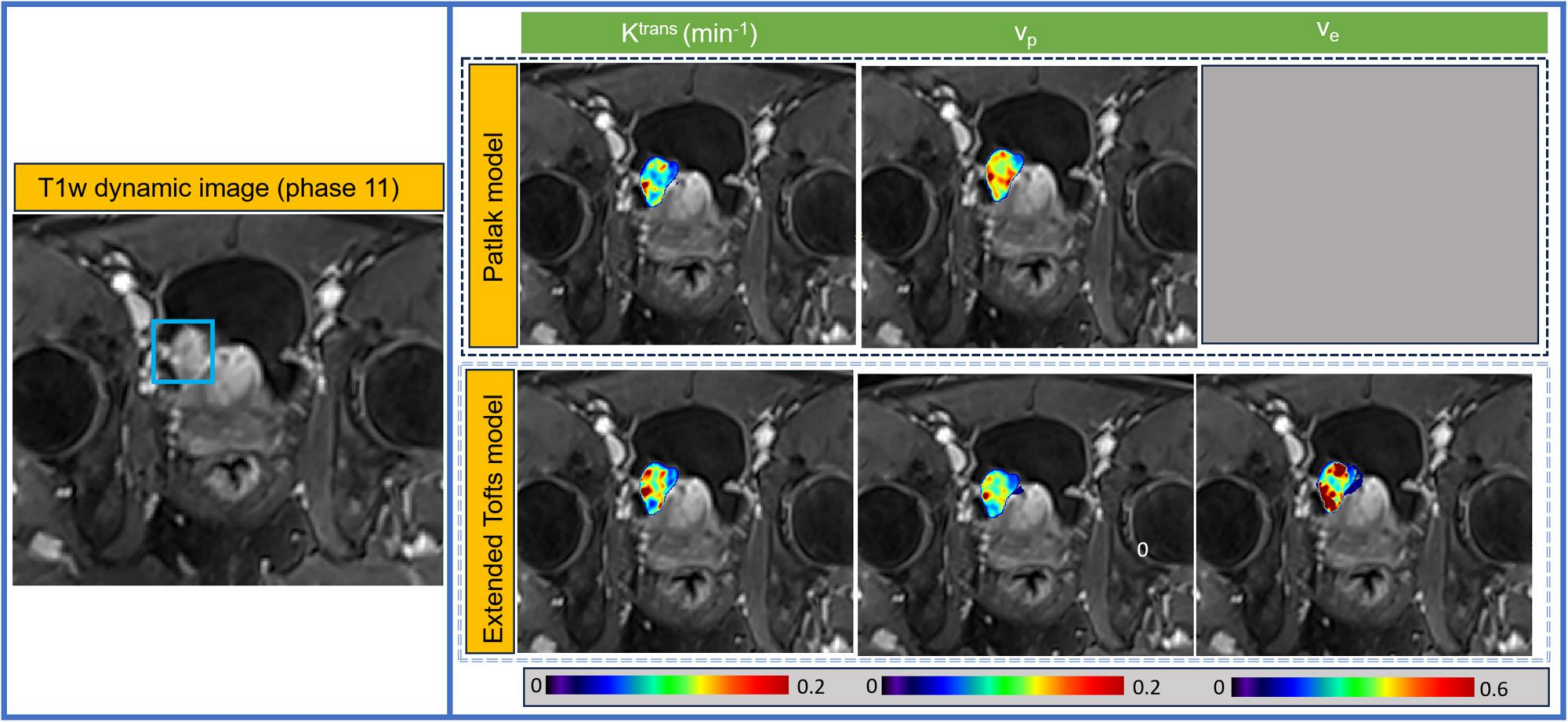
MRI-MBA (Model-Based Analysis) includes algorithms to analyse DCE-MRI (dynamic contrast-enhanced) data using Patlak and extended Toft models. These models derived representative parametric maps are overlaid on the post-contrast T1-weighted images.

Figure 5 shows the representative parametric maps of the parameters, which were generated from the Patlak model (*K*^trans^ and *v_p_*) and the extended Tofts model (*K*^trans^, *v_e_*, and *v_p_*), respectively.

The mean (± SD) values of the extended Tofts model-derived *K*^trans^, *v_e_*, and *v_p_* were 0.27 ± 0.22 (min^−1^), *v_e_* = 038 ±0.19, and *v_p_* = 0.03 ± 0.02, respectively. For the Patlak model, *K*^trans^ and *v_p_* values were 0.076 ± 0.075 (min^−1^) and 0.079 ± 0.073, respectively. These QIBs were extracted from NMIBC patients (*n* = 34) prior to treatment. The mean (±SD) *K*^trans^, *v*_e_, and *v*_p_ values derived from the extended Tofts model for pure UC (*n* = 31) and npVH (*n* = 3) were as follows: 0.28 ± 0.23 vs. 0.19 ± 0.09, 0.40 ± 0.19 vs. 0.20 ± 0.16, and 0.0243 ± 0.0205 vs. 0.020 ± 0.008, respectively. The results highlight the utility of MRI-MBA, which provides valuable QIBs for clinical applications in BCa.

## Discussion

There is a growing demand for advanced quantitative imaging tools with AI-driven models to address specific clinical endpoints.^7^^,^^52^ Currently, available tools for quantitative analysis of mpMRI data are still closed-source, limiting their broader adoption and utilization.^1–4^^,^^18^ Unlike these existing solutions that address bladder-specific challenges, our AI-BLADE is the first bladder-specific tool for analysing mpMRI data. It has exhibited its capabilities to perform tasks such as histological pattern recognition, predictive analytics, and real-time monitoring that can be used easily by both clinicians and imaging researchers. The toolbox enabled the exploration of different AI and machine learning algorithms and configurations, allowing for a thorough evaluation of model performance across a variety of settings, as well as the generation of QIBs’ numerical and parametric maps.

Recent studies highlighted the importance of deep features extracted from intermediate or FC layers of CNNs, as these capture hierarchical and abstract patterns in imaging data beyond what handcrafted features can achieve.^39^^,^^53^ Zhu et al compared “off-the-shelf” deep features extracted from DCE-MRI data using networks like GoogleNet and VGG with transfer learning approaches for breast cancer subtype classification, achieving the highest AUC of 0.65 with pretrained deep feature extraction.^54^ Kang et al^55^ expanded this approach by extracting deep features from 13 pretrained CNNs (eg, VGG, ResNet, Inception) on brain tumour MRI data and combining them using an ensemble of machine learning models. Their best performance configuration used an SVM with a radial basis function kernel, resulting in a significant improvement in classification performance. Similarly, Du et al^56^ fused deep features from multicontrast MRI (T1w, T2w, DW-MRI) data via a custom deep network to stratify high-grade serous and clear cell ovarian cancers into their distinct subtypes, achieving an AUC of 0.916. In the present BCa study, we pair deep feature extraction from T2w imaging data with classical classifiers to form a robust and interpretable framework. VGG19 combined with a Decision Tree classifier and no feature selection achieved the highest performance (AUC = 0.79). The EfficientNet-B7 demonstrated strong performance (AUC = 0.73) without feature selection. Similarly, ResNet152 and AlexNet consistently yielded lower AUCs, regardless of feature selection or classifier choice. Extracted features were assessed both with and without feature selection prior to classifier training.

The strong performance observed in the BCa classification with VGG19 can be attributed to its sequential convolutional design using small 3 × 3 filters and deeper hierarchical layers, which enhance feature abstraction and spatial detail capture within bladder lesions. Unlike architectures such as DenseNet or EfficientNet that rely on dense connectivity or compound scaling, VGG19 provides a more stable feature extraction process, reducing the risk of overfitting in small and heterogeneous datasets. These findings suggest that moderately deep, uniform convolutional architectures may offer a favourable balance between generalization and discriminative power for bladder mpMRI analysis. Emerging as the most effective feature extractor, VGG19 underscores the potential of the MRI-DFA toolkit as a generalizable framework for broader clinical applications by leveraging pretrained deep learning architectures and integrating feature selection strategies. We anticipate further testing of the MRI-DFA toolkit for different oncological endpoints.

Quantitative DW- and DCE-MRI, through their derived QIBs, together interrogate tumour physiology by evaluating cellularity and cell membrane integrity via water diffusion, and vascular permeability and integrity using CA, respectively.^7^

The degree of DW-MRI signal attenuation caused by restricted or hindered water diffusion depends on the changes in EES volume and increased tissue tortuosity. Areas of restricted diffusion appear as high signal intensity on DW images and correspond to low ADC values on ADC maps. These imaging characteristics provide diagnostic and prognostic insights, such as tumour stage, grade, and size, correlated with histopathological findings.

Bladder tumours typically exhibit higher cellular density, leading to lower ADC values than normal bladder wall (1.05 ±0.22 × 10^−^³ mm^2^/s vs. 1.83 ± 0.18 × 10^−^³ mm^2^/s).^57^ Sevcenco et al reported that MIBC exhibited significantly lower ADC values than the NMIBC (0.759 × 10^−3^mm^2^/s vs. 1.120 × 10^−3^ mm^2^/s).^58^ Similarly, Li et al reported lower ADC values for MIBC of 0.964 ×10^−^³ mm^2^/s and compared to NMIBC 1.205 ×10^−^³ mm^2^/s (the highest *b*-value of 2000 s/mm^2^).^17^ In the present study, the ADC value of 1.22 × 10^−^³ mm^2^/s for the NMIBC cohort is within the range of reported values.^13^

ADC is a composite metric that accounts for both diffusion within the EES and the capillary network, which follows a Gaussian distribution for water diffusion. The deviation of diffusivity from a non-Gaussian distribution that arises from tissue microstructural complexity. The curvature observed in signal decay at higher *b*-values is effectively captured by the diffusion kurtosis imaging (DKI) model, characterized by QIB, *D*_app_, and *K*_app_.^7^ Li et al used DKI-derived parameters to stratify MIBC and NMIBC; for MIBC, *D*_app_ values were 1.635 ×10^−3^ (mm^2^/s) and *K*_app_ = 0.78, respectively, whereas for NMIBC these values were 2.038 ×10^−3^ (mm^2^/s) and 0.610, respectively.^17^ To date, no study has reported the QIBs value derived from the NG-IVIM model to the best of our knowledge. NG-IVIM estimates “K” in addition to the true molecular diffusion in the EES and perfusion-related QIBs in the capillary network (ie, *f* and D*). In the present study, the *K* value (derived from NG-IVIM rather than the DKI model) was 0.41, slightly lower than the one reported in the above study. This could be due to different data modelling approaches, as well as the use of different multiple *b*-value DW data acquisition protocols.

The DCE-MRI signal enhancement pattern exhibited is closely reflected by tumour vascular integrity. For example, NMIBC tumours, which are typically smaller and more superficial, with small areas of necrosis, often exhibit functionality of the tumour vasculature, contributing to higher contrast enhancement.^59^ The semi-quantitative analysis of DCE data can extract parameters such as time-intensity curves, wash-in/wash-out rates, and AUC.^60^ These quantities have demonstrated potential for tumour staging and grading^60^ but lack physiological specificity and do not directly quantify tumour physiological QIBs, such as *K*^trans^, which reflects both blood flow and vascular permeability, with its value depending on the tissue characteristics.^19^ To our knowledge, this is the first study to apply the Patlak model in BCa, yielding *K*^trans^ and *v_p_* values of 0.076 min^−1^ and 0.075, respectively. The Patlak model is simpler, linear, computationally efficient, and less sensitive to noise compared to nonlinear models like the Tofts model. It also accounts for irreversible CA extravasation from plasma into the EES without backflux, suitable for quantifying low levels of CA extravasation. However, for significant backflux, a full reversible 2-compartment model, the extended Tofts model, may be necessary to quantify and maintain the quantitative accuracy of QIBs. Nash et al used the extended Tofts model in patients with histologically confirmed BCa (stage T2-T4) and reported the values of *K*^trans^ = 0.085 min^−1^, *v_e_* = 0.34, and *v_p_* = 0.0095.^61^ In the present study, these corresponding values for the NMIBC cohort derived from the extended Tofts model were 0.27 min^−1^, 0.38, and 0.03, respectively. The mean *K*^trans^, *v_p_*, and *v_e_* values showed a trend toward differences of 33%, 49%, and 17%, respectively, between pure UC (*n* = 31) and those with npVH (*n* = 3) among NMIBC patients. The mean *K*^trans^ value is within the range of the reported value^13^; however, these QIBs’ numerical value may vary, depending on the data acquisition protocol, signal quality, the specific models used for data fitting, and the tumour tissue under study. We anticipate further validation of the MRI-MBA toolkit through the acquisition of more DW- and DCE-MRI data for clinical endpoints, such as classification of histology subtypes.

The final component of the toolbox, shown in the workflow, allows users to perform stepwise feature selection and classification towards various clinical endpoints, followed by statistical analysis. This adaptability in feature selection implies that the AI-BLADE toolbox can be effectively utilized across a broad spectrum of clinical applications. The combination of different biomarkers provides an understanding of tumour biology, facilitating more personalized treatment options. The AI-BLADE toolbox has the potential to impact both imaging research and clinical practice in BCa significantly.

**Figure 6.**
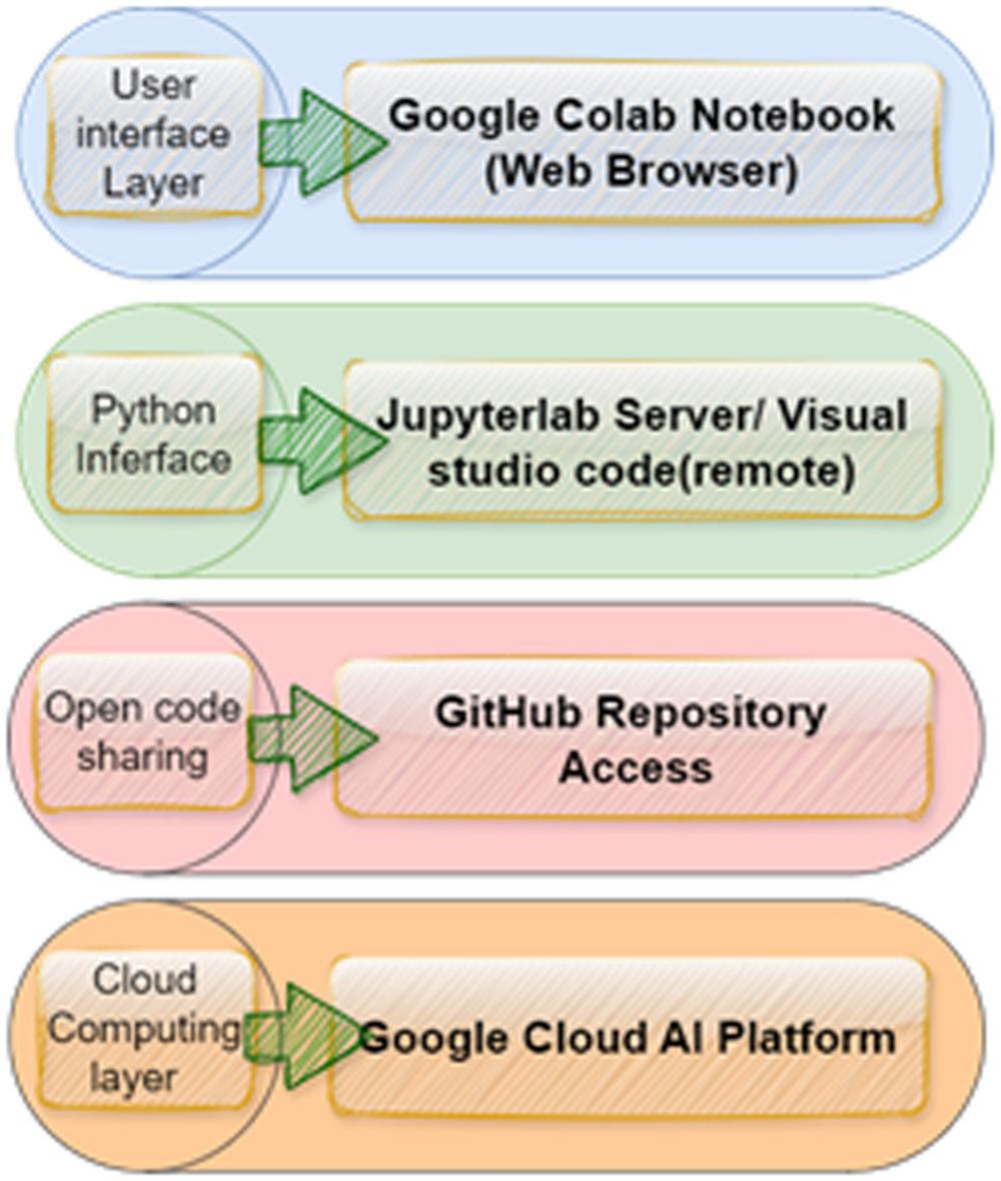
Proposed architecture for web-based cloud implementation of AI-BLADE (AI-powered BLADdEr multiparametric MRI Analysis for Clinical Application).

Currently, AI-BLADE can be made available through a material transfer agreement with our centre. Our future work will focus on automating the ROI annotation and tumour segmentation process using advanced deep learning–based algorithms within the AI-BLADE V2.0 framework, enabling a fully integrated and scalable clinical workflow. We plan to make AI-BLADE accessible via web-based cloud architectures for broader availability and use (Figure 6).

## Conclusion

The AI-BLADE (v1.0), a flexible and user-friendly software toolbox for analysing mpMRI data, shows strong potential for application in BCa oncology, offering capabilities that can enhance diagnostic accuracy and support improved patient outcomes. The MRI-DFA toolkit integrates novel deep learning models with advanced feature selection methods to improve diagnostic accuracy, speed, and interpretability in clinical research. In parallel, the MRI-MBA toolkit derives physiological QIBs through data-driven modelling of DWand DCE data, enabling characterization of the tumour microenvironment. Together, deep features and QIBs offer complementary insights into the tumour microenvironment that can enhance the diagnosis, monitoring, and treatment of BCa, particularly in clinical settings where timely decision-making is critical for effective patient management.

## Supplementary Material

ubag002_Supplementary_Data
