# Supplementary material for: AI-BLADE toolbox: AI-powered BLADdEr multiparametric MRI analysis for clinical application
Source: BJR Artif Intell. 2026 Jan 22;3(1):ubag002. doi: 10.1093/bjrai/ubag002 (PMC13045516; doi:10.1093/bjrai/ubag002)
Supplement: ubag002_Supplementary_Data [file ubag002_supplementary_data.zip › Appendix A_Clean.docx]

**Appendix** **A**.

Table 1. Bladder MRI protocol

| **Parameter** | **T_1_w** | **T_2_w** | **DW** | **DCE** |
| --- | --- | --- | --- | --- |
| **Field Strength** | 1.5 T/3T | 1.5 T/3T | 1.5 T/3T | 1.5 T/3T |
| **Sequence** | FRFSE | FRFSE | SS-EPI | FSPGR |
| Plane Orientation | Multiplanar | Multiplanar | Axial | Axial |
| Field of view (FOV) (mm) | 250-350 | 250-350 | 200-250 | 200-250 |
| Repetition time (TR) (ms) | 400-700 | 4000-6000 | 3500-5000 | 3.5-4.5 |
| Echo time (TE) (ms) | 1.2-2.4 | 82-120 | 60-80 | 1.2-2.2 |
| **Acquisition Matrix** | 256-320 x 192-256 | 256-320 x 192-256 | 128 x 128 | 256 x 192 |
| Slice thickness/gap (mm) | 3-4 / 1-0 | 3-4 / 1-0 | 4-0 / 1-0 | 4-0 / 1-0 |
| Number of excitations | 2 | 2 | 2-8 | 1 |
| **Flip Angles (FAs) (deg)** |  |  |  | 15 |
| # b-values (s/mm^2^) | N/A | N/A | 0 and 800-1000, up to 2000 optional | N/A |

T1w–T1 weighted imaging, T2w-T2 weighted imaging, DW–Diffusion weighted, DCE-Dynamic contrast enhanced

T_10_ mapping acquisition: FAs: 5^0^, 15^0^, and 30^0^; Other MR parameters are the same as DCE acquisition


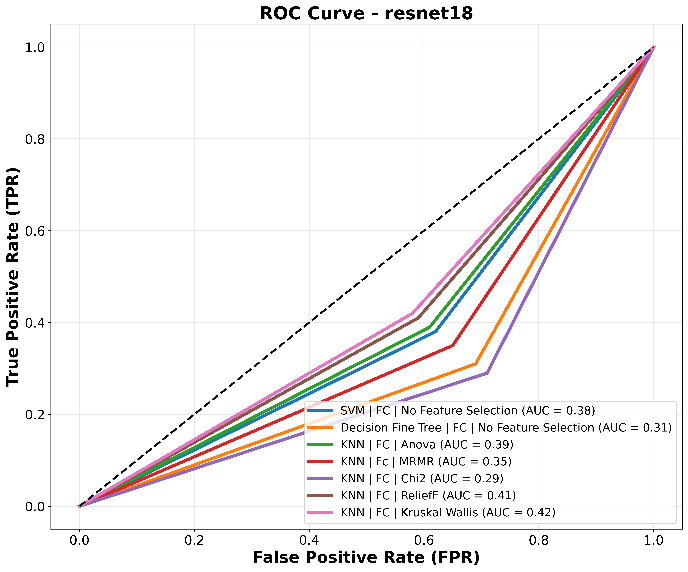

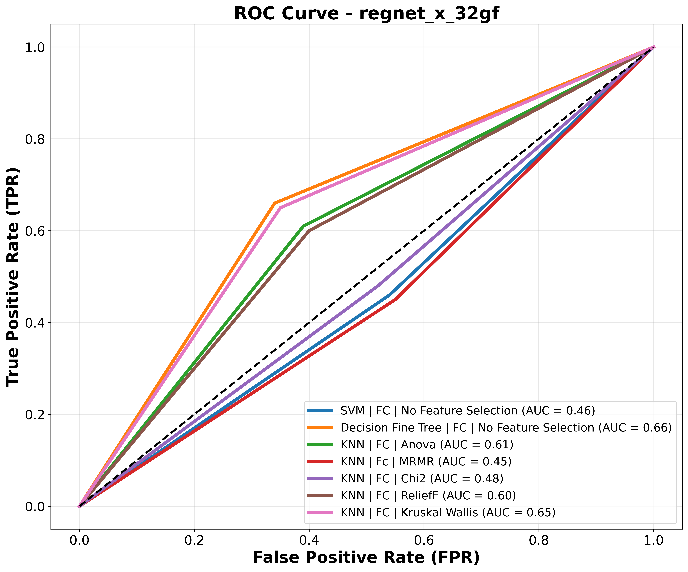

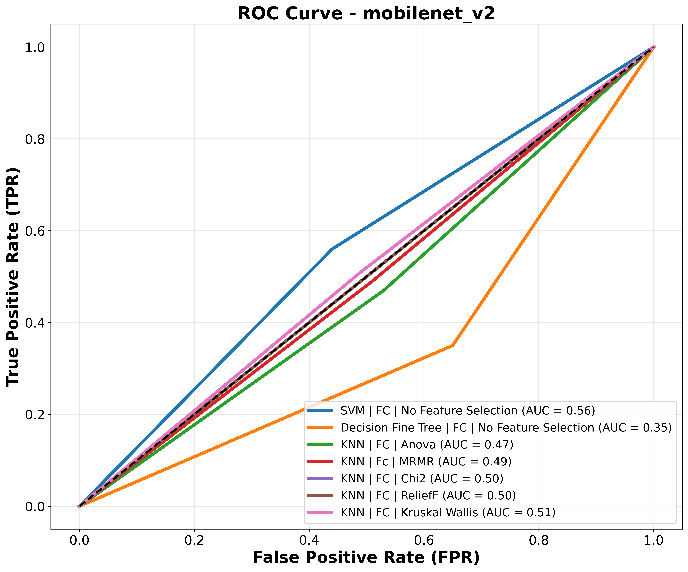

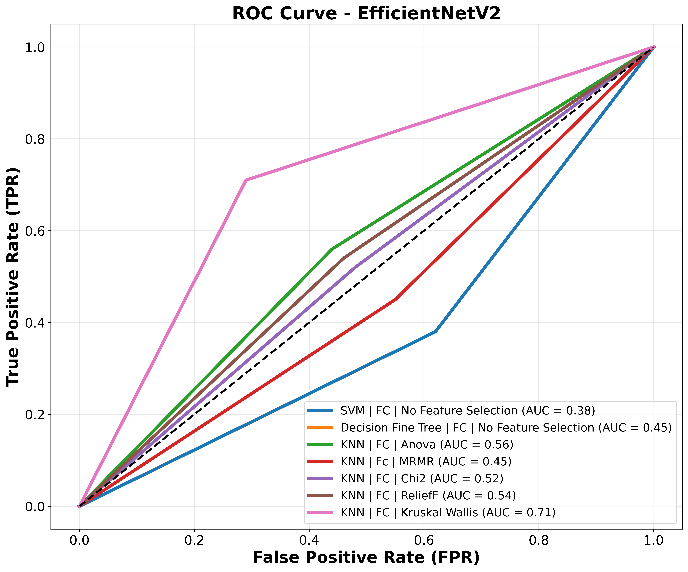

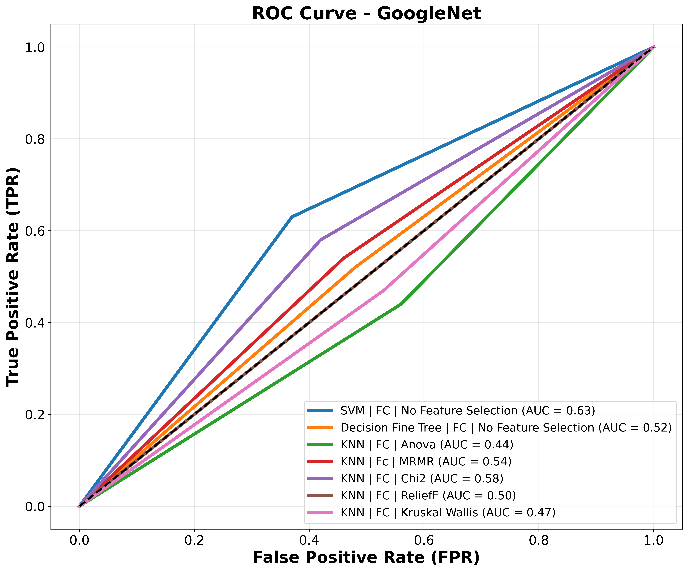

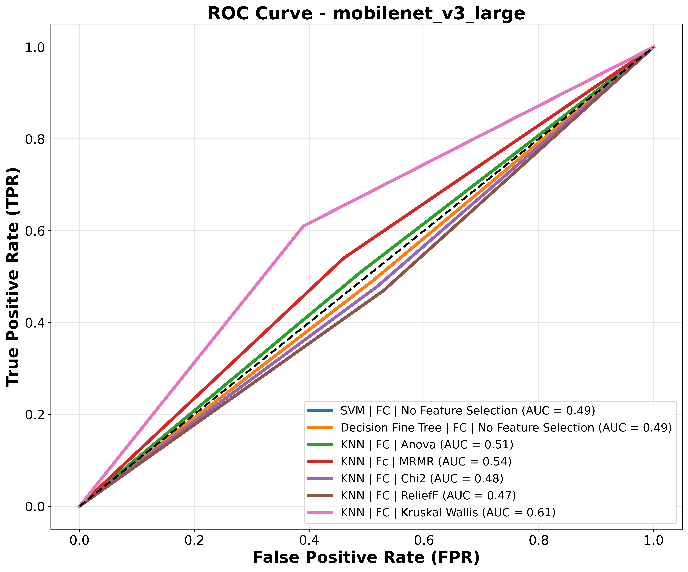


f

e

d

c

b

a


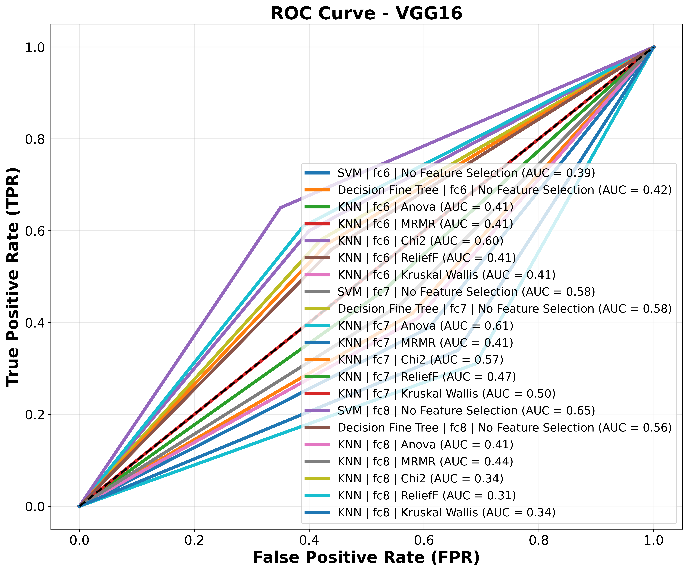

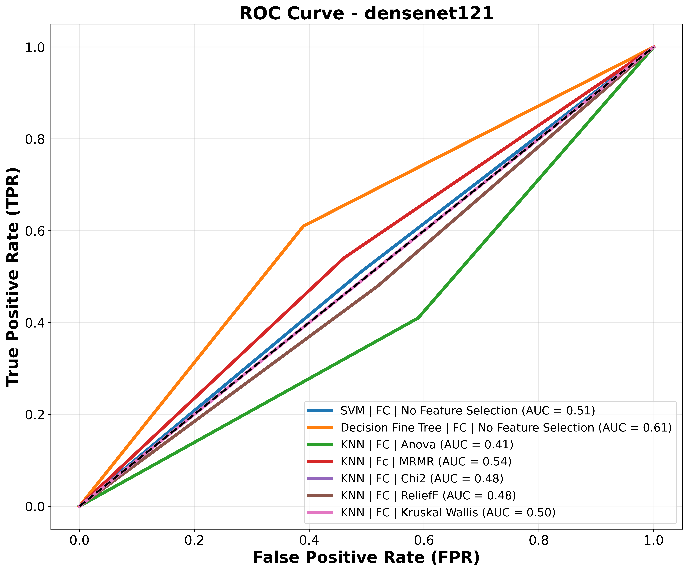

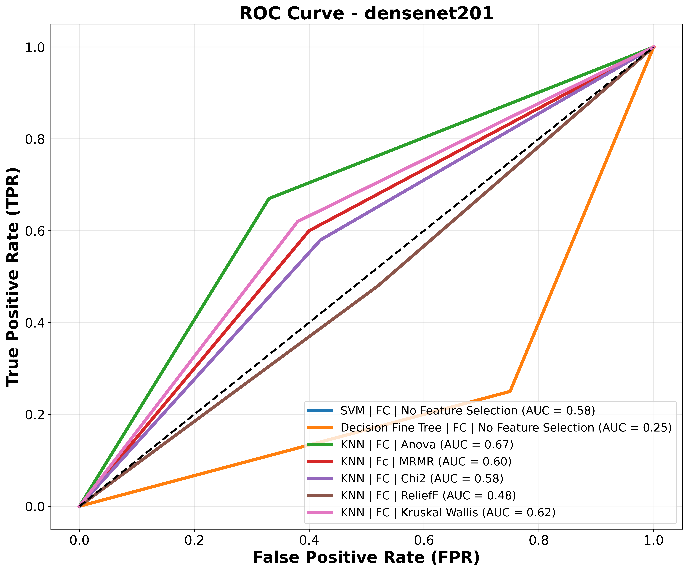


i

j

h

g

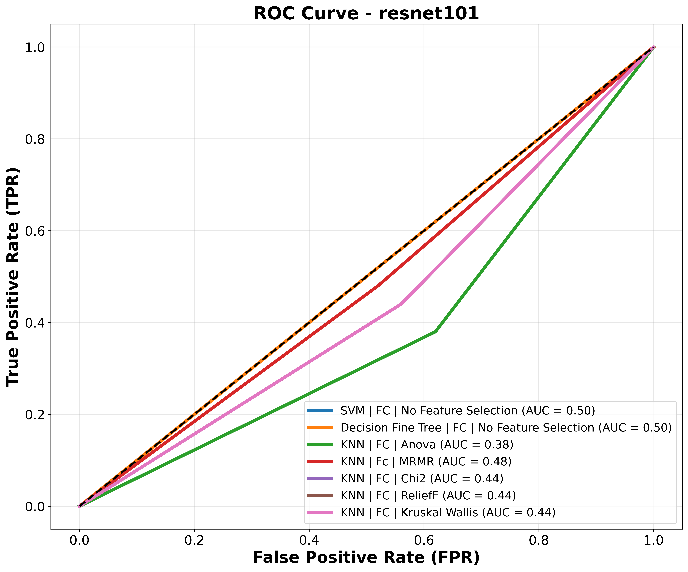


Figure 1: *ROC curve for AI models (a-j) using the MRI-DFA toolkit with different feature selection algorithms and classifiers*
